# Association Between the Use of a Digital Health Platform During Pregnancy and Helping Users Avoid Emergency and In-Person Care: Retrospective Observational Study

**DOI:** 10.2196/43180

**Published:** 2023-05-15

**Authors:** Hannah R Jahnke, Lily Rubin-Miller, Natalie Henrich, Christa Moss, Neel Shah, Alex Peahl

**Affiliations:** 1 Maven Clinic New York, NY United States; 2 Harvard Medical School Boston, MA United States; 3 Department of Obstetrics and Gynecology, Beth Israel Deaconess Medical Center Boston, MA United States; 4 Department of Obstetrics and Gynecology University of Michigan Ann Arbor, MI United States

**Keywords:** pregnancy, telehealth, web-based care, emergency room, value-based care, digital care, prenatal care

## Abstract

**Background:**

Almost one-third of pregnant people visit the emergency room during pregnancy. Although some emergency care is necessary, gaps in patient education and inaccessibility of preventive services have been identified as key reasons for high-cost, low-value care in pregnancy. Digital platforms present a promising solution for providing resources to supplement routine prenatal care, thereby reducing the use of low-value in-person services.

**Objective:**

This study aimed to describe the relationship between the use of Maven and in-person care avoidance (emergency room or office visits) during pregnancy. Maven is a digital prenatal health platform that supplements routine prenatal care. Maven offers educational content (articles, videos, and classes), care coordination (through a care advocate), and provider services (web-based appointments and communication with providers) designed to complement prenatal care. Specifically, the aims of this study were to examine whether the use of Maven is associated with in-person care avoidance overall and whether improvements in pregnancy-related knowledge facilitate in-person care avoidance. To assess aim 2, we tested if the use of Maven is associated with improvements in self-reported understanding of warning signs and medically accurate information and if self-reported understanding of medically accurate information and warning signs is associated with in-person care avoidance in a population of Maven users.

**Methods:**

In this retrospective study, we used adjusted logistic regression to examine the relationship between digital platform use, avoidance of in-person care, and the platform’s influence on pregnancy-related knowledge (learning medically accurate information and recognizing warning signs). Demographics, medical history, and in-person care avoidance were self-reported.

**Results:**

Of the 5263 users, 280 (5.32%) reported that Maven helped them avoid in-person care during pregnancy. More users who reported avoiding in-person care also reported that the digital platform helped them understand warning signs (231/280, 82.5%) and learned medically accurate information (185/280, 66.1%). In the adjusted models, all modes of digital service use (assessed as quartiles) were associated with avoiding in-person care in a dose-response manner (eg, web-based provider appointments: Q2 adjusted odds ratio [aOR] 1.57, 95% CI 1.00-2.41; Q3 aOR 2.53, 95% CI 1.72-3.72; Q4 aOR 5.26, 95% CI 3.76-7.42). Users were more likely to avoid in-person care if they reported that Maven helped them recognize warning signs (aOR 3.55, 95% CI 2.60-4.94) or learn medically accurate information (aOR 2.05, 95% CI 1.59-2.67).

**Conclusions:**

These results suggest that digital platforms can be effective in helping patients to avoid in-person care. The educational pathway suggests that digital platforms can be particularly effective in helping patients recognize warning signs and learn medically accurate information, which may help them avoid in-person care by recognizing when in-person care is medically appropriate. Future work is needed to assess other pathways through which digital resources can support pregnant people and improve perinatal care use.

## Introduction

Nonurgent emergency room (ER) use among pregnant people is common [1], contributing to high-cost, low-value care. Together, gaps in patient education and the inaccessibility of preventive services have been identified as key reasons for ER presentation during pregnancy [2]. A recent study found that among patients who went to the ER during their pregnancy, over one-third reported that they did so because they either lacked access to care from nonemergency providers or lacked the knowledge to address and evaluate their needs. Furthermore, 45% of pregnant people in the study went to the ER because they were concerned that there was an emergency, and they could not get the answers they needed in a timely fashion from their physician [2]. Although leading maternity care organizations have called for improved patient education and counseling on warning signs [3,4], several barriers prevent their implementation in real-world practice, including time, provider availability, and patient-centered resources.

Digital platforms are a promising approach for providing resources and services that may fill gaps in existing prenatal patient education and care delivery systems. Specifically, digital platforms could provide opportunities to reduce activities or facilities of low-value in-person care that could be appropriately and safely replaced with information and coaching through many pathways, including providing pregnant people with a web-based option for information and support. Furthermore, digital platforms can eliminate traditional barriers to care, allowing health education and web-based care to be more accessible, affordable, and comprehensive [5]. Although telehealth presents a potential solution for filling educational gaps in routine prenatal care [6], to date, little is known about how prenatal digital health platforms may influence patient education and in-person care use.

Maven is a comprehensive digital platform for women’s and family health that was developed to provide support services that supplement and complement routine prenatal care. Users receive free and unlimited access to Maven as an employer-sponsored health benefit through their own or their partner’s employer. On the digital platform, Maven offers educational content (articles, videos, and live classes), care coordination (through a dedicated care advocate), and provider services (web-based appointments and communication with a diverse team of providers). Maven services do not replace routine prenatal care.

**Figure 1:**
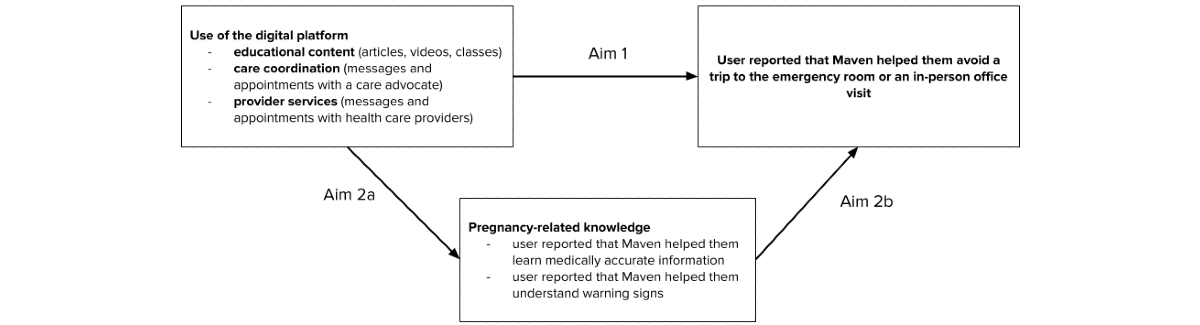
Conceptual model (digital platforms can help reduce in-person care through several pathways. Here, we assessed how an educational pathway may contribute to this reduction).

Although there are many reasons for using nonroutine in-person care during pregnancy, digital resources are particularly well positioned to help users avoid unnecessary in-person care by building pregnancy-related knowledge and digitally offering access to providers. Knowledge can help patients differentiate routine symptoms of pregnancy from more concerning warning signs, thereby preventing unnecessary presentations for in-person care. For example, a pregnant person may learn that round ligament pain is normal during pregnancy, allowing them to avoid in-person assessment of the expected symptoms. This retrospective study investigated how the use of Maven may help pregnant people avoid ER or in-person appointments (hereafter, *in-person* is used to refer to either ER or other in-person care) during pregnancy and how patient education may serve as a mechanism for this outcome. Specifically, the aims of this study were to examine (1) if the use of a digital health platform is associated with in-person care avoidance overall and (2) how pregnancy-related knowledge may serve as one of the mechanisms for this association. To assess aim 2, we tested (1) how the use of a digital health platform is associated with a better understanding of warning signs and medically accurate information and (2) how understanding medically accurate information and warning signs is associated with in-person care avoidance in a population of Maven users. These pathways are illustrated in a conceptual model (Figure 1).

## Methods

### Study Design and Setting

This retrospective cohort analysis assessed the association between user engagement with a digital health platform and user reports that the platform helped them avoid in-person care. Users received access to the digital platform as an employer-sponsored health benefit through their own or their partner’s employer, or through their health plan. We included users who were enrolled in the program during their pregnancy between January 2020 and July 2022 and delivered at the time of data analysis (July 2022). Users were included if they completed health surveys at both onboarding to the pregnancy program (onboarding survey) and the postpartum program (postdelivery survey), and their zip code could be mapped to the Social Vulnerability Index (SVI).

### Ethical Consideration, Informed Consent, and Participation

The study protocol was designated as exempt by the WCG Institutional Review Board, an independent ethical review board. All users consented to the use of their deidentified data for scientific research upon creating an account. All data used in this analysis were deidentified.

### Data Collection

All use data were tracked using the digital platform. Demographic and health data were self-reported on the onboarding survey, and outcome data and additional health data were self-reported on the postdelivery survey.

The use of the digital platform included three types of services: (1) *educational content*, (2) *care coordination*, and (3) *provider services*. First, *educational content* refers to the educational content available on the digital platform included web-based classes, class recordings, and articles. Users have access to various articles, classes, and videos related to pregnancy, birth, and the postpartum period. Example articles included “First trimester warning signs to watch for,” “Your anatomy scan, explained,” and “4 mental exercises to combat pregnancy anxiety.” In web-based classes, users can engage with a health care provider in a group setting to learn and ask questions. Example classes include “Childbirth education 101” and “How to manage stress and anxiety.” Web-based classes were also recorded and uploaded to the digital health platform for users to watch asynchronously as video content. Measures of educational content included the number of articles read, number of class recordings watched, and number of live web-based classes attended, all of which were assessed in quartiles. Second, *care coordination* includes interactions with a user’s dedicated care advocate. On this platform, care advocates are allied health professionals (eg, nurses and social workers) who serve as users’ primary point of contact within the digital platform, support the coordination of digital prenatal services, and direct users to providers and services (both web based and in-person when necessary). We assessed the number of messages sent by users to their care advocates in quartiles. We also assessed whether users had at least 1 care advocate appointment, as users were encouraged to have 1 introductory appointment with a care advocate upon enrollment, and most members communicated with their care advocate through messages thereafter. Third, *provider services* were assessed through interactions with health care providers, including obstetrician/gynecologists (OB/GYNs), doulas, midwives, mental health providers, nutritionists, wellness coaches, and others. We assessed the number of messages sent by users to providers and the number of appointments with providers in quartiles. We also assessed whether users had at least one appointment with an OB/GYN or a mental health provider, as we hypothesized that web-based appointments with these 2 types of care providers may be particularly beneficial for helping users avoid in-person care. Appointments with these providers were assessed dichotomously as 0 or one or more appointments, based on the distribution of data. The pregnancy trimester of enrollment, assessed categorically, was used as a proxy for an individual’s time enrolled in the digital platform.

The primary study outcome was whether a user reported that the digital care platform helped them avoid in-person care on the postdelivery survey. To assess this outcome, users were asked, “In what way(s) did Maven influence your experience?” and the response options included “Maven helped me avoid a trip to the ER or an in-person office visit.” Users who selected this option were recorded as yes, and users who did not select this option were recorded as no.

Self-report of whether the digital platform helped users understand warning signs during pregnancy and learn medically accurate information about pregnancy or its complications was assessed in the postdelivery survey. To assess these responses, users were asked, “Did Maven help you understand warning signs during pregnancy?” with response options of yes or no, and “In what way(s) did Maven influence your experience?” where users were offered the option to select “Maven helped me learn medically accurate information” as one of a variety of response options. Users who selected this option were recorded as yes, and users who did not select this option were recorded as no.

User demographics and health data were self-reported. The age at onboarding was calculated internally from the user’s date of birth. Race and ethnicity were asked in the same question, and race was categorized into Hispanic or Latine and non-Hispanic or Latine: White, Asian or Pacific Islander, Black, and Other (which was composed of multiracial and American Indian users). Users were asked to select from a list of medical conditions all those that applied to them. Medical conditions were then assessed in statistical models as a risk score for the outcome, which was calculated by adding the number of medical conditions reported by each user. This approach was informed by a literature review that found that regardless of which chronic diseases an adult was diagnosed with, an individual was more likely to have an ER visit as their number of chronic diseases increased [7], and that the number of chronic conditions is associated with health anxiety [8], which is in turn associated with higher health care use [8,9]. Therefore, we combined chronic conditions into a single summative score on an interval scale to control for potential health conditions, with a higher score indicating an increasing number of conditions. This method has been used extensively in medical research [8,10]. Medical conditions used to calculate the risk score included chronic conditions (eg, heart disease, diabetes, high blood pressure, blood disorder, thrombophilia, kidney disease, thyroid disease, autoimmune disease, HIV or AIDS, and obesity) and current pregnancy-related conditions (eg, cholestasis, fetal growth restriction, gestational hypertension, preeclampsia, and gestational diabetes). Mental health conditions were aggregated into 1 dichotomous variable for any mental health condition versus no mental health condition. Mental health conditions included history of anxiety, depression, and perinatal mood disorders. Pregnancy-related anxiety was assessed on a 5-item Likert scale in response to “On a scale of 1-5, how anxious are you feeling about your pregnancy?” with responses of 4 (“very”) or 5 (“extremely”) indicating the presence of pregnancy-related anxiety.

We used the Centers for Disease Control and Preventions’ SVI as a proxy for social determinants of health. SVI is a geographic measure of a community’s vulnerability that incorporates data from 4 domains: socioeconomic status, household composition and disability, minority status and language, and housing type and transportation [11]. The SVI is assigned to each user based on the zip code. To convert user zip codes to census tract–based SVI, we used a 2020 weighted crosswalk from the US Department of Housing and Urban Development [12,13]. In the analyses, SVI was dichotomized into low (≤0.80) and high (>0.80), with high representing the highest risk quintile nationally.

### Statistical Analysis

We conducted descriptive analyses to assess the relationship between users’ characteristics and the report of the digital platform to help users avoid in-person care. In bivariate analyses, chi-square or Fisher exact tests were used to assess categorical variables, and 2-tailed *t* tests and Mann-Whitney *U* tests were used to assess continuous variables.

Adjusted logistic regression was used to assess the relationship within each aim. Covariates were selected because they were considered confounders for each model based on directed acyclic graphs [14]. We chose to control for members’ health characteristics in aim 1 but not in aim 2, based on which variables we considered true confounders in each analysis. In aim 1, we believed that a user’s medical conditions may cause differential use of digital resources (exposure) and differential use of in-person care (outcome); therefore, we included it as a confounder. For aim 2a, although a user’s medical conditions likely cause differential use of digital resources (exposure), we were not confident that their medical conditions would cause them to learn more or less from the platform (outcome); someone’s medical conditions do not affect their capacity to learn. Similarly, for aim 2b, we were not confident that someone’s medical condition affects their ability to learn from Maven (exposure). Controlling for fields that are not true confounders introduces bias within the causal inference theory [14]; thus, we did not control for medical conditions in the statistical models for aim 2.

For aim 1, assessing the association between digital prenatal platform use and the perceived influence of the digital platform on in-person care avoidance, each use metric was assessed in its own model. Adjusted logistic regression models controlled for age, race and ethnicity, medical risk score, mental health conditions, pregnancy-related anxiety, parity, pregnancy trimester enrolled, and high SVI. For aim 2a, assessing how use of the digital platform is associated with a better understanding of medically accurate information and warning signs, adjusted models controlled for parity and high SVI. For aim 2b, assessing how understanding medically accurate information and warning signs is associated with the perceived influence of the digital platform on in-person care avoidance, adjusted models controlled for parity, high SVI, and pregnancy trimester enrolled.

All analyses were conducted in R (version 3.6.3; R Foundation for Statistical Computing) [15].

## Results

### Descriptive Statistics

**Figure 2:**
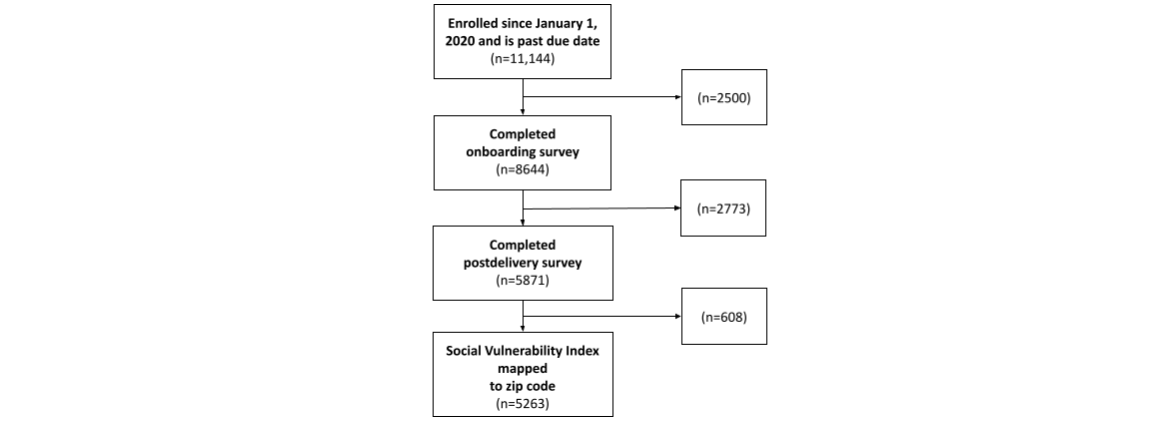
Flow diagram for study eligibility.

The final sample included 5263 users who used the product during pregnancy and reported outcomes after delivery (Figure 2). The average age of the sample was 32.7 (SD 4.0) years (Table 1). Most of the sample were White (2200/5263, 41.8%), and 18.2% (958/5263) were Asian or Pacific Islander, 7.9% (416/5263) were Hispanic, 4.5% (237/5263) were Black, and 2.3% (121/5263) were multiracial or American Indian (Other). Many participants preferred not to disclose their racial and ethnic identity (1332/5263, 25.31%). Additionally, 2.91% (153/5263) of the sample had a high SVI. Furthermore, 11.61% (611/5263) of the sample had pregnancy-related anxiety, 26.11% (1374/5263) had a history of at least 1 mental health condition, whereas most had no medical conditions (3174/5263, 60.3%).

Among the included users, 5.3% (280/5263) reported that the digital platform helped them avoid in-person care, 56.39% (2968/5263) reported that it helped them understand warning signs, and 47.9% (2521/5263) reported that it helped them learn medically accurate information during pregnancy (Table 2).

Users who reported avoiding an in-person care visit were more likely to be younger (*P*<.001), be people of color or Hispanic (*P*<.001), have pregnancy-related anxiety (*P*=.03), and have a mental health condition (*P*<.001; Table 1). They were also more likely to have enrolled in the digital platform earlier in their pregnancy (*P*<.001); use more digital services in each category (*P*<.001); and report that the digital health platform influenced the way they approached their maternity experience (*P*<.001), helped them understand warning signs (*P*<.001), helped them learn medically accurate information about pregnancy and complications (*P*<.001), and helped them manage their anxiety and depression (*P*<.001). Of those who avoided in-person care, 82.5% (231/280) reported that the digital platform helped them learn warning signs and 66.1% (185/280) reported that the digital platform helped them learn medically accurate information (Table 2).

Articles were the most used resource on the digital platform, with use ranging from 0 to 8, 9 to 22, 23 to 48, and 49 to 399 article reads in ascending quartiles. Approximately, 69.14% (3639/5263) of users had at least 1 appointment with a care advocate, 19.86% (1045/5263) of users had at least 1 appointment with an OB/GYN, and 8.89% (468/5263) of users had at least 1 appointment with a mental health provider during pregnancy (Table 2).

**Table 1:** User characteristics by emergency department or in-person avoidance.

					Maven helped me avoid a trip to the emergency room or an in-person office visit						Overall (N=5263)			*P* value	
					No (n=4983)			Yes (n=280)							
**Demographics**															
	Age (years), mean (SD)			32.8 (3.99)			31.9 (3.91)			32.7 (3.99)			<.001		
	Nulliparous, n (%)			3682 (73.89)			205 (73.21)			3887 (73.86)			.80		
	**Race and ethnicity, n (%)**													<.001	
		Asian or Pacific Islander		905 (18.16)			52 (18.57)			957 (18.18)					
		Black		213 (4.27)			25 (8.93)			238 (4.52)					
			Hispanic			387 (7.77)			31 (11.07)			418 (7.94)			
		White		2103 (42.2)			95 (33.93)			2198 (41.76)					
		Other^a^		111 (2.23)			9 (3.21)			120 (2.28)					
		I prefer not to say		1264 (25.37)			68 (24.29)			1332 (25.31)					
	High Social Vulnerability Index, n (%)			140 (2.81)			11 (3.93)			151 (2.87)			.27		
**Medical conditions, n (%)**															
	**Chronic conditions**														
		Heart disease		23 (0.46)			0 (0)			23 (0.44)			.63		
		Diabetes		56 (1.12)			7 (2.5)			63 (1.2)			.05		
		High blood pressure		168 (3.37)			12 (4.29)			180 (3.42)			.41		
		Blood disorder		52 (1.04)			1 (0.36)			53 (1.01)			.53		
		Thrombophilia		29 (0.58)			0 (0)			29 (0.55)			.40		
		Kidney disease		10 (0.2)			1 (0.36)			11 (0.21)			.45		
		Thyroid disease		384 (7.71)			20 (7.14)			404 (7.68)			.73		
		Autoimmune disease		151 (3.03)			7 (2.5)			158 (3)			.61		
		HIV or AIDS		1 (0.02)			0 (0)			1 (0.02)			.99		
		Obesity		793 (15.91)			48 (17.14)			841 (15.98)			.59		
	**Pregnancy-related conditions**														
		Cholestasis		68 (1.36)			6 (2.14)			74 (1.41)			.29		
		Fetal growth restriction		128 (2.57)			5 (1.79)			133 (2.53)			.42		
		Gestational hypertension		623 (12.5)			40 (14.29)			663 (12.6)			.38		
		Preeclampsia		287 (5.76)			15 (5.36)			302 (5.74)			.78		
		Gestational diabetes		517 (10.38)			32 (11.43)			549 (10.43)			.57		
	**Medical risk score^b^**													.56	
		0		2996 (60.12)			176 (62.86)			3172 (60.27)					
		1		1225 (24.58)			60 (21.43)			1285 (24.42)					
		2		497 (9.97)			23 (8.21)			520 (9.88)					
		3		191 (3.83)			13 (4.64)			204 (3.88)					
		4		57 (1.14)			6 (2.14)			63 (1.2)					
		5		14 (0.28)			1 (0.36)			15 (0.29)					
		6		3 (0.06)			1 (0.36)			4 (0.08)					
	**Mental health conditions^c^**														
		Depression		620 (12.44)			41 (14.64)			661 (12.56)			.28		
		Anxiety		1137 (22.82)			84 (30)			1221 (23.2)			.01		
		Perinatal mood disorder		101 (2.03)			11 (3.93)			112 (2.13)			.03		
	Pregnancy-related anxiety			569 (11.42)			44 (15.71)			613 (11.65)			.03		

^a^Other includes multiracial and American Indian.

^b^Sum of chronic conditions and pregnancy-related conditions.

^c^Variable has missingness (<6% of sample).

**Table 2:** Use and user-reported outcomes by emergency department or in-person avoidance.

				Maven helped me avoid a trip to the emergency room or an in-person office visit, n (%)					Overall (N=5263), n (%)			*P* value
				No (n=4983)		Yes (n=280)						
**Use**												
	**Pregnancy trimester enrolled**											<.001
		First trimester	1243 (24.94)		101 (36.07)		1344 (25.54)					
		Second trimester	2147 (43.09)		129 (46.07)		2276 (43.25)					
		Third trimester	1593 (31.97)		50 (17.86)		1643 (31.22)					
**Educational content**												
	**Articles read^a^**											<.001
		Q1: [0,8]	1342 (26.93)		35 (12.5)		1377 (26.16)					
		Q2: (8,22]	1252 (25.12)		57 (20.36)		1309 (24.87)					
		Q3: (22,48]	1206 (24.2)		71 (25.436)		1277 (24.26)					
		Q4: (48,399]	1183 (23.74)		117 (41.79)		1300 (24.7)					
	**Class recordings watched^a^**											<.001
		0	2573 (51.64)		113 (40.36)		2686 (51.04)					
		(0,3]	1408 (28.26)		78 (27.86)		1486 (28.23)					
		(3,103]	1002 (20.1)		89 (31.79)		1091 (20.73)					
	**Web-based classes attended^a^**											.001
		0	2722 (54.62)		124 (44.29)		2846 (54.08)					
		(0,2]	1170 (23.48)		72 (25.71)		1242 (23.6)					
		(2,18]	1091 (21.89)		84 (30)		1175 (22.32)					
**Care coordination**												
	**Messages sent to care advocate^a^**											<.001
		0	2016 (40.46)		69 (24.64)		2085 (39.6)					
		(0,1]	700 (14.05)		30 (10.71)		730 (13.9)					
		(1,5]	1196 (24)		71 (25.36)		1267 (24.1)					
		(5,81]	1071 (21.49)		110 (39.29)		1181 (22.4)					
	At least 1 care advocate appointment		3429 (68.81)		210 (75)		3639 (69.14)			.03		
**Provider services**												
	**Messages sent to providers^a^**											<.001
		0	3184 (63.9)		99 (35.36)		3283 (62.4)					
		(0,2]	745 (14.95)		56 (20)		801 (15.2)					
		(2,196]	1054 (21.15)		125 (44.64)		1179 (22.4)					
	**Total web-based provider appointments attended^a^**											<.001
		(0)	2397 (48.1)		63 (22.5)		2460 (46.74)					
		(0,1]	856 (17.18)		34 (12.14)		890 (16.91)					
		(1,3]	872 (17.5)		57 (20.36)		929 (17.65)					
		(3,117]	858 (17.22)		126 (45)		984 (18.7)					
	**At least 1 appointment with**											
		Obstetrician/gynecologist providers	905 (18.16)		140 (50)		1045 (19.86)			<.001		
		Mental health providers	419 (8.41)		49 (17.5)		468 (8.89)			<.001		
**User-reported outcomes**												
	Something I learned through Maven influenced the way I approached my maternity experience^b^		2938 (58.96)		251 (89.64)		3189 (60.59)			<.001		
	Maven helped me understand warning signs during pregnancy^b^		2737 (54.93)		231 (82.5)		2968 (56.39)			<.001		
	Maven helped me learn medically accurate information about pregnancy and complications		2336 (46.88)		185 (66.07)		2521 (47.9)			<.001		
	Maven helped me manage anxiety and depression		554 (11.12)		85 (30.36)		639 (12.14)			<.001		

^a^Shown in quartiles.

^b^Variable has missingness (<6% of sample).

### Effect of Digital Platform Use on Avoidance of In-Person Care

In adjusted models, higher use of all educational content, care coordination, and provider services were significantly associated with higher odds of avoiding in-person care in a dose-response manner (Table 3). First- and second-trimester enrollment were associated with higher odds (adjusted odds ratio [aOR] 2.49, 95% CI 1.74-3.61 and aOR 1.86, 95% CI 1.32-2.65, respectively) of avoiding in-person care than third-trimester enrollment. Among the educational content, reading more articles had the greatest effect on avoiding in-person care (Q2: aOR 1.73, 95% CI 1.11-2.73; Q3: aOR 2.27, 95% CI 1.45-3.60; and Q4: aOR 3.98, 95% CI 2.55-6.33), followed by watching more video recordings and attending more web-based classes. A similar dose response was observed across all use categories. Regarding care coordination, sending more messages to care advocates (Q3: aOR 1.69, 95% CI 1.19-2.41; and Q4: aOR 2.73, 95% CI 1.97-3.82) and having at least 1 web-based appointment with a care advocate (aOR 1.42, 95% CI 1.07-1.91) were associated with avoiding an in-person visit. Regarding provider services, attending more web-based provider appointments (Q2: aOR 1.57, 95% CI 1.00-2.41; Q3: aOR 2.53, 95% CI 1.72-3.72; and Q4: aOR 5.26, 95% CI 3.76-7.42) and sending more messages to providers were both associated with avoiding an in-person visit in a dose-response manner. When broken down by provider type, both having at least 1 web-based appointment with an OB/GYN (aOR 4.19, 95% CI 3.21-5.47) and having at least 1 web-based appointment with a mental health provider (aOR 2.07, 95% CI 1.45-2.89) were associated with avoiding in-person care (Table 3).

**Table 3:** Effect of web-based care use on avoidance in-person care (N=5263).

					Unadjusted						Adjusted			
					Odds ratio (95% CI)			*P* value			Adjusted odds ratio (95% CI)^a^			*P* value
**Pregnancy trimester enrolled**														
	First trimester			2.59 (1.84-3.69)			<.001			2.49 (1.74-3.61)			<.001	
	Second trimester			1.91 (1.38-2.69)			<.001			1.86 (1.32-2.65)			<.001	
	Third trimester			Ref^b^			N/A^c^			Ref			N/A	
**Educational content**														
	**Articles read^d^**													
		Q1	Ref			N/A			Ref			N/A		
		Q2	1.75 (1.14-2.70)			.01			1.73 (1.11-2.73)			.01		
		Q3	2.26 (1.51-3.44)			<.001			2.27 (1.45-3.60)			<.001		
		Q4	3.79 (2.61-5.65)			<.001			3.98 (2.55-6.33)			<.001		
	**Class recordings watched^d^**													
		Q1 and Q2	Ref			N/A			Ref			N/A		
		Q3	1.26 (0.94-1.69)			.12			1.22 (0.89-1.68)			.02		
		Q4	2.02 (1.51-2.69)			<.001			2.00 (1.44-2.77)			<.001		
	**Web-based classes attended^d^**													
		Q1 and Q2	Ref			N/A			Ref			N/A		
		Q3	1.35 (1.00-1.81)			.05			1.31 (0.95-1.80)			.1		
		Q4	1.69 (1.27-2.24)			<.001			1.67 (1.20-2.31)			.02		
**Care coordination**														
	**Messages sent to care advocate^d^**													
		Q1	Ref			N/A			Ref			N/A		
		Q2	1.25 (0.80-1.92)			.3			1.25 (0.78-1.95)			.3		
		Q3	1.73 (1.24-2.44)			.001			1.69 (1.19-2.41)			.004		
		Q4	3.00 (2.21-4.11)			<.001			2.73 (1.97-3.82)			<.001		
	At least 1 care advocate appointment			1.36 (1.04-1.80)			.03			1.42 (1.07-1.91)			.02	
**Provider services**														
	**Messages sent to care providers^d^**													
		Q1 and Q2	Ref			N/A			Ref			N/A		
		Q3	2.42 (1.72-3.37)			<.001			2.34 (1.64-3.31)			<.001		
		Q4	3.81 (2.91-5.02)			<.001			3.35 (2.50-4.50)			<.001		
	**Total web-based provider appointments attended^d^**													
		Q1	Ref			N/A			Ref			N/A		
		Q2	1.65 (1.10-2.44)			.01			1.57 (1.00-2.41)			.04		
		Q3	2.69 (1.89-3.81)			<.001			2.53 (1.72-3.72)			<.001		
		Q4	5.70 (4.24-7.72)			<.001			5.26 (3.76-7.42)			<.001		
	**At least 1 appointment with**													
		Obstetrician/gynecologist providers	4.51 (3.53-5.76)			<.001			4.19 (3.21-5.47)			<.001		
		Mental health providers	2.31 (1.65-3.17)			<.001			2.07 (1.45-2.89)			<.001		

^a^Models were adjusted for age, race and ethnicity, medical risk score, mental health conditions, pregnancy-related anxiety, parity, pregnancy trimester enrolled, and high Social Vulnerability Index.

^b^Ref: reference group.

^c^N/A: not applicable.

^d^Shown in quartiles.

### Effect of Digital Platform Use on Pregnancy-Related Knowledge

A similar dose-response effect for each type of use was observed in adjusted models predicting the odds of helping users understand warning signs during pregnancy and learn medically accurate information about pregnancy and its complications (Table 4). In every adjusted model, more use of educational content, care coordination, and provider services was significantly associated with higher odds of the outcome. Overall, first-trimester enrollers were more likely to report understanding warning signs (aOR 3.40, 95% CI 2.92-3.97) and learn medically accurate information (aOR 2.47, 95% CI 2.13-2.87) when compared with third-trimester enrollers. Among the educational content, reading more articles had the greatest effect on understanding warning signs (Q2: aOR 2.00, 95% CI 1.71-2.34; Q3: aOR 3.48, 95% CI 2.96-4.09; and Q4: aOR 6.46, 95% CI 5.42-7.71) and learning medically accurate information (Q2: aOR 2.07, 95% CI 1.76-2.44; Q3: aOR 3.39, 95% CI 2.88-4.01; and Q4: aOR 5.61, 95% CI 4.72-6.67). A similar dose-response effect was observed for sending messages to a care advocate, sending messages to providers, and attending appointments with providers. Having at least one appointment with a care advocate, an OB/GYN, and a mental health provider also affected these outcomes (Table 4).

**Table 4:** Effect of use on Maven helping users understand warning signs in pregnancy and learn medically accurate information about pregnancy (N=5263).

				Maven helped me understand warning signs during pregnancy									Maven helped me learn medically accurate information about pregnancy and complications						
				OR^a^ (95% CI)		*P* value		aOR^b^ (95% CI)^c^		*P* value		OR (95% CI)			*P* value		aOR (95% CI)^c^		*P* value
**Pregnancy trimester enrolled**																			
	First trimester		3.47 (2.98-4.04)		<.001		3.40 (2.92-3.97)		<.001		2.54 (2.19-2.95)			<.001		2.47 (2.13-2.87)		<.001	
	Second trimester		2.19 (1.93-2.50)		<.001		2.16 (1.89-2.46)		<.001		1.85 (1.62-2.11)			<.001		1.80 (1.58-2.06)		<.001	
	Third trimester		Ref^d^		N/A^e^		Ref		N/A		Ref			N/A		Ref		N/A	
**Educational content**																			
	**Articles read^f^**																		
		Q1	Ref		N/A		Ref		N/A		Ref			N/A		Ref		N/A	
		Q2	2.04 (1.74-2.38)		<.001		2.00 (1.71-2.34)		<.001		2.16 (1.84-2.55)			<.001		2.07 (1.76-2.44)		<.001	
		Q3	3.57 (3.04-4.19)		<.001		3.48 (2.96-4.09)		<.001		3.61 (3.07-4.26)			<.001		3.39 (2.88-4.01)		<.001	
		Q4	6.78 (5.72-8.06)		<.001		6.46 (5.42-7.71)		<.001		6.23 (5.27-7.37)			<.001		5.61 (4.72-6.67)		<.001	
	**Class recordings watched^f^**																		
		Q1 and Q2	Ref		N/A		Ref		N/A		Ref			N/A		Ref		N/A	
		Q3	1.96 (1.72-2.23)		<.001		1.86 (1.64-2.13)		<.001		2.22 (1.95-2.53)			<.001		2.08 (1.83-2.37)		<.001	
		Q4	2.94 (2.53-3.43)		<.001		2.70 (2.31-3.15)		<.001		3.66 (3.15-4.25)			<.001		3.26 (2.81-3.80)		<.001	
	**Web-based classes attended^f^**																		
		Q1 and Q2	Ref		N/A		Ref		N/A		Ref			N/A		Ref		N/A	
		Q3	1.71 (1.49-1.96)		<.001		1.61 (1.40-1.84)		<.001		1.67 (1.46-1.91)			<.001		1.53 (1.34-1.76)		<.001	
		Q4	2.66 (2.30-3.08)		<.001		2.40 (2.06-2.79)		<.001		2.92 (2.54-3.37)			<.001		2.53 (2.18-2.93)		<.001	
**Care coordination**																			
	**Messages sent to care advocate^f^**																		
		Q1	Ref		N/A		Ref		N/A		Ref			N/A		Ref		N/A	
		Q2	1.06 (0.90-1.26)		.50		1.06 (0.90-1.26)		.50		1.1 (0.93-1.31)			.30		1.1 (0.93-1.31)		.30	
		Q3	1.49 (1.29-1.71)		<.001		1.47 (1.27-1.69)		<.001		1.69 (1.47-1.95)			<.001		1.66 (1.44-1.92)		<.001	
		Q4	2.45 (2.11-2.86)		<.001		2.32 (1.99-2.70)		<.001		2.97 (2.56-3.45)			<.001		2.76 (2.38-3.21)		<.001	
	At least 1 care advocate appointment		1.12 (1.07-1.19)		<.001		1.30 (1.16-1.47)		<.001		1.34 (1.19-1.50)			<.001		1.34 (1.19-1.51)		<.001	
**Provider services**																			
	**Messages sent to care providers^f^**																		
		Q1 and Q2	Ref		N/A		Ref		N/A		Ref			N/A		Ref		N/A	
		Q3	1.8 (1.53-2.11)		<.001		1.77 (1.50-2.08)		<.001		1.93 (1.65-2.26)			<.001		1.9 (1.62-2.22)		<.001	
		Q4	2.13 (1.85-2.45)		<.001		2.07 (1.80-2.39)		<.001		2.48 (2.17-2.85)			<.001		2.42 (2.11-2.78)		<.001	
	**Total web-based provider appointments attended^f^**																		
		Q1	Ref		N/A		Ref		N/A		Ref			N/A		Ref		N/A	
		Q2	1.14 (0.98-1.33)		.09		1.15 (0.98-1.34)		.08		1.37 (1.17-1.60)			<.001		1.38 (1.18-1.62)		<.001	
		Q3	1.73 (1.49-2.03)		<.001		1.66 (1.43-1.95)		<.001		2.39 (2.05-2.79)			<.001		2.29 (1.96-2.67)		<.001	
		Q4	2.53 (2.16-2.96)		<.001		2.44 (2.08-2.87)		<.001		3.10 (2.66-3.63)			<.001		2.98 (2.55-3.49)		<.001	
	**At least 1 appointment with**																		
		Obstetrician/gynecologist providers	2.12 (1.83-2.45)		<.001		2.04 (1.76-2.37)		<.001		2.22 (1.93-2.55)			<.001		2.12 (1.84-2.44)		<.001	
		Mental health providers	1.35 (1.11-1.65)		.002		1.42 (1.17-1.74)		<.001		1.18 (0.98-1.43)			.84		1.25 (1.03-1.52)		.02	

^a^OR: odds ratio.

^b^aOR: adjusted odds ratio.

^c^Models were adjusted for high Social Vulnerability Index and parity.

^d^Ref: reference group.

^e^N/A: not applicable.

^f^Shown in quartiles.

### Effect of Pregnancy-Related Knowledge on Avoidance of In-Person Care

In the adjusted models, understanding warning signs and learning medically accurate information from the digital platform were independently associated with in-person care avoidance (aOR 3.55, 95% CI 2.60-4.94 and aOR 2.05, 95% CI 1.59-2.67, respectively, with N=5263 and *P* values <.001). Models were adjusted for high SVI, parity, and pregnancy trimester enrolled.

## Discussion

### Principal Findings

The results of this retrospective study suggest that patient education, care coordination, and provider services offered through a comprehensive digital platform present promising solutions for improving pregnancy-related knowledge and reducing low-value ER use and in-person care for pregnant patients.

These results suggest that digital platforms can provide information and tools that patients need to recognize warning signs, avoid medical misinformation, and decide when in-person care is medically appropriate during pregnancy. This study found that longer durations of digital platform use and greater use of all digital services, including all facets of educational content, care coordination, and provider services, were associated with helping users avoid ER or in-person care by understanding warning signs in their pregnancies and learning medically accurate information. A dose-response effect was observed for all uses assessed in quartiles. Reading articles and attending web-based provider appointments were the most predictive of helping users avoid ER or in-person care. Attending at least one appointment with an OB/GYN and attending at least one appointment with a mental health provider were also independently associated with helping users to avoid ER or in-person care. Reading articles, watching class recordings, and attending web-based appointments were most predictive of users’ reporting that the platform helped them understand warning signs and learn medically accurate information. Reports that the digital platform helped users understand warning signs and learn medically accurate information were associated with helping users avoid ER or in-person care.

### Comparison With Prior Work

Our analysis found that the use of resources on a digital platform was associated with user reports of learning medically accurate information and understanding warning signs, which in turn were associated with helping pregnant people avoid in-person care. Leaders in maternity care have advocated patient education as a key way to mitigate pregnancy-related morbidity and mortality. A recent report from maternal mortality review committees found that over 80% of pregnancy-related deaths were preventable [16], and other review committees found that lack of knowledge on warning signs was among the most common contributors to maternal mortality [4]. Furthermore, the Centers for Disease Control and Preventions’ Hear Her campaign aims to prevent pregnancy-related deaths by increasing awareness of pregnancy-related complications and their warning signs [3]. Our results suggest that digital technology can be used to help pregnant people learn medically accurate information and recognize warning signs, which could contribute to more timely identification and management of pregnancy complications.

In addition to helping patients recognize warning signs for physical health conditions, education and support through digital platforms can be useful in helping them manage their mental health. Previous studies have found that increased knowledge of pregnancy complications from digital health platforms is associated with a reduction in maternal anxiety [17]. Not only is mental health management through digital health platforms beneficial to patients’ mental health directly but also it has the ability to improve physical health [18] and help decrease inappropriate in-person care use [19,20]. Studies have found that untreated mental health conditions, including depression and anxiety during and after pregnancy, are associated with worse maternal and infant health, leading to higher use of services and health care costs [21]. As such, digital platforms have the potential to fill gaps in education, as well as preventive and mental health services, presenting a promising solution for mitigating the use of low-value in-person care.

Promoting high-value care by supplementing routine care with access to a digital platform is beneficial for patients, payers, and health systems. For patients, the benefits of telehealth include ease of use, improved communication with providers, after-hours care, timely information, high patient satisfaction, and decreased travel and wait times [22-24]. The literature on patient satisfaction reports that digital health users generally report high confidence in and acceptance of digital health, which can result in more patient activation and education [25]. Furthermore, digital solutions can decrease the stress of navigating in-person care, particularly during ER visits. Telehealth can also expand the accessibility of resources by overcoming geographic and logistical barriers [26] and can have substantial cost savings for patients from avoided childcare and transportation, promoting health equity [27].

With the rise of accountable care organizations and the use of value-based payment [28], patients avoiding unnecessary ER and in-person visits provide opportunities for cost savings for health systems and payers [29]. Furthermore, avoiding unnecessary ER and in-person care opens up capacity in hospitals and health centers to more effectively treat patients with urgent needs [30,31]. Potential value extends beyond care efficiency: increased pregnancy-related knowledge gained through digital health platforms may drive downstream improvements for other health outcomes [32], including mental health and maternal and infant outcomes [17], which could drive cost savings for the health care system while improving population health [19,20].

### Strengths and Limitations

This study extends the available literature by assessing the potential influence of access to comprehensive digital health services on ER and in-person care avoidance during pregnancy. Our study has several strengths. We assessed a national sample of commercially insured pregnant people who were not limited to a single health care system. Second, we leveraged product use data that are tracked internally, so they are not subject to recall bias.

This study also has limitations. First, except for use data, all data were self-reported by users, which may cause misreporting of medical conditions and a subjective report of whether the digital platform was influential in avoiding an ER or in-person care. However, this method also allows for an in-depth assessment of users’ experiences and perspectives, which provides a unique, patient-centered perspective on the value of digital solutions. Second, this analysis did not account for the simultaneous use of various types of web-based resources throughout pregnancy, as these are not necessarily confounders (it is unclear how the use of one resource influences the use of another); thus, their inclusion could introduce bias [33]. Future research could consider how the total use of digital resources across resource types as a score or latent variable is associated with these outcomes. Third, this retrospective study used data designed to meet multiple needs: research and outcome evaluation, clinical care, and product improvement. Consequently, data collection was not optimized solely for research purposes, resulting in a lack of specificity in some of our data fields. For example, here we examined whether users report that they were helped to avoid in-person care at all, but it is not possible to assess which kinds of care were avoided, and it does not disaggregate ER from other in-person care. In addition, we cannot assess the ways in which digital health services help patients avoid in-person care; for example, users may have preferred digital services or found them more accessible. Future research should investigate the specific types of care that digital services are best suited to help users avoid. Finally, these data were collected during the peak of the COVID-19 pandemic, a time when people may have sought to avoid in-person care more actively. Future research is needed to assess whether these effects differ in more diverse populations, including patients with public insurance, greater social vulnerability, and historically marginalized racial and ethnic groups.

### Conclusions

Multifaceted digital health platforms such as Maven can help reduce the use of in-person care, including ER visits during pregnancy. Digital platforms are well-suited for providing users with convenient, high-quality, and patient-centered educational materials and web-based care that can improve pregnancy-related knowledge and reduce in-person care, which can be costly and burdensome.

## Abbreviations

aOR: adjusted odds ratio
ER: emergency room
OB/GYN: obstetrician/gynecologist
SVI: Social Vulnerability Index
